# Using Advanced Spectroscopy and Organic Matter Characterization to Evaluate the Impact of Oxidation on Cyanobacteria

**DOI:** 10.3390/toxins11050278

**Published:** 2019-05-17

**Authors:** Saber Moradinejad, Caitlin M. Glover, Jacinthe Mailly, Tahere Zadfathollah Seighalani, Sigrid Peldszus, Benoit Barbeau, Sarah Dorner, Michèle Prévost, Arash Zamyadi

**Affiliations:** 1 BGA Innovation Hub and Civil, Mineral and Mining Engineering Department, Polytechnique Montréal, Montréal, QC H3T 1J4, Canada; saber.moradinejad@polymtl.ca (S.M.); caitlinmeara@gmail.com (C.M.G.); jacinthe.mailly@polymtl.ca (J.M.); tahere.zadfathollah-seighalani@polymtl.ca (T.Z.S.); benoit.barbeau@polymtl.ca (B.B.); sarah.dorner@polymtl.ca (S.D.); michele.prevost@polymtl.ca (M.P.); 2 Department of Civil & Environmental Engineering, University of Waterloo, Waterloo, ON N2L 3G1, Canada; speldszus@uwaterloo.ca

**Keywords:** cyanobacteria, oxidation, cell morphology, enhanced darkfield microscopy/hyperspectral imaging, intracellular organic matter

## Abstract

Drinking water treatment plants throughout the world are increasingly facing the presence of toxic cyanobacteria in their source waters. During treatment, the oxidation of cyanobacteria changes cell morphology and can potentially lyse cells, releasing intracellular metabolites. In this study, a combination of techniques was applied to better understand the effect of oxidation with chlorine, ozone, potassium permanganate, and hydrogen peroxide on two cell cultures (*Microcystis, Dolichospermum*) in Lake Champlain water. The discrepancy observed between flow cytometry cell viability and cell count numbers was more pronounced for hydrogen peroxide and potassium permanganate than ozone and chlorine. Liquid chromatography with organic carbon and nitrogen detection was applied to monitor the changes in dissolved organic matter fractions following oxidation. Increases in the biopolymer fraction after oxidation with chlorine and ozone were attributed to the release of intracellular algal organic matter and/or fragmentation of the cell membrane. A novel technique, Enhanced Darkfield Microscopy with Hyperspectral Imaging, was applied to chlorinated and ozonated samples. Significant changes in the peak maxima and number of peaks were observed for the cell walls post-oxidation, but this effect was muted for the cell-bound material, which remained relatively unaltered.

## 1. Introduction

Rising temperatures and the eutrophication of freshwaters have contributed to an increase in the frequency of cyanobacterial blooms [1,2,3]. Cyanobacterial cells are of concern because they can produce and release cyanotoxins as well as taste and odor (T&O) compounds. T&O compounds affect the aesthetic water quality, result in customer complaints, and lower the trust customers have in their water [4]. If ingested, cyanotoxins can cause gastroenteritis, cytotoxicity, liver damage, neurological effects and they have been linked to cancer, Alzheimer’s disease and Motor Neuron Disease [5,6]. To protect customers, regulatory agencies have proposed and, in some cases, set health-based guidelines and threshold alert levels for both cells and their metabolites [7,8,9]. 

The removal of cyanobacteria and their metabolites is often a challenge for conventional water treatment (i.e., coagulation, flocculation, sedimentation and filtration) with both cyanotoxins and/or T&O compounds observed in finished drinking water [10,11,12]. However, cyanobacteria can also breakthrough filtration in water treatment plants considered to be of low risk, i.e., with low intake cell concentrations in their source waters, and high-risk, i.e., during bloom events [10,13,14]. Cyanobacterial breakthrough, particularly for low-risk plants, has been attributed to the accumulation of cells during treatment, e.g., in filter beds, in the sludge bed of sedimentation tanks, and in sludge thickeners [10,13,15]. Apart from the breakthrough of cyanobacteria and their metabolites, the presence of intracellular and extracellular organic matter (IOM and EOM) from algae, increases the coagulant demand and the formation of disinfection byproducts [16].

A number of studies have evaluated the oxidation (e.g., ozone, chlorine, potassium permanganate, and hydrogen peroxide) of cyanobacteria and their metabolites [17,18,19,20,21]. The impact of these oxidants ranges from cell damage to the release of metabolites through diffusion or cell lysis; the level of impact varies depending on the species of cyanobacteria present, presence of a slime layer, and the background water matrix, i.e., pH and background organic matter [19,20,22,23]. To model the release of metabolites, previous work has applied pseudo first-order sequential reaction rates to track the release of intracellular metabolites and the subsequent oxidation of the extracellular metabolites, i.e., intracellular—rate of release—extracellular—rate of oxidation—oxidized metabolites [24,25]. However, when model results have been compared with full-scale oxidation data, they often overestimate the oxidation efficiency [17,24,26]. In addition, the release of cyanotoxins and/or T&O compounds can occur at very low doses (e.g., at detection limit for chlorine), making it very difficult to assess the potential risk during treatment [27,28]. 

To better understand the changes in morphology that may be causing the discrepancy between expected release and actual release, previous work has used both Scanning Electron Microscopy (SEM) and Digital Flow Cytometry [20,29,30,31,32]. However, these techniques are qualitative and do not provide quantitative results. Enhanced Darkfield Microscopy with Hyperspectral Imaging (EDM/HSI) not only captures images of the sample, but also measures the visible wavelength spectrum (400–1000 nm) of each pixel. The black background in EDM allows the instrument to collect scattered light from the desired (i.e., cells) pixels of a sample. HSI has been used successfully to identify cyanobacteria blooms in laboratory and remote sensing applications [33,34,35,36]; however, it has not yet been applied to evaluate the impact of oxidation on cyanobacteria cells. 

In this study, a combination of techniques was used to explore the impact of oxidation on cyanobacteria cells. The objectives of this study were as follows: (1) to quantify changes of two cyanobacteria in natural water following oxidation with chlorine, ozone, hydrogen peroxide, and potassium permanganate with flow cytometry, SEM, and liquid chromatography with organic carbon and nitrogen detection (LC-OCD-OND) and (2) to compare these results against the morphological changes observed with EDM/HSI after oxidation with chlorine and ozone. To the best of the author’s knowledge, this study presents the first assessment of the morphological deformation of cyanobacteria after oxidation with EDM/HSI.

## 2. Results and Discussion

### 2.1. Cell Viability Post-Oxidation

**Figure 1:**
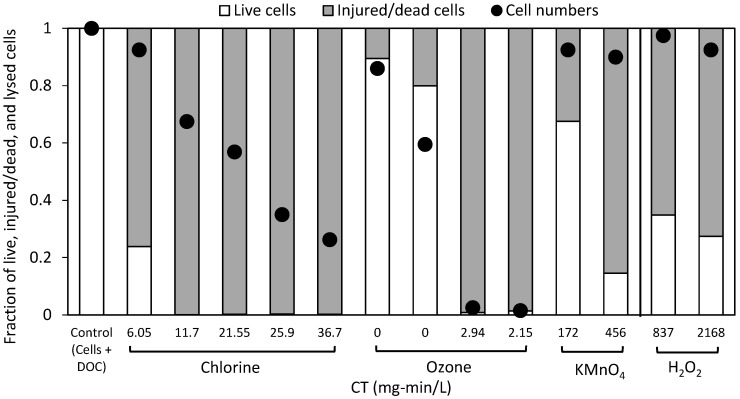
Impact of chlorine, ozone, potassium permanganate, and hydrogen peroxide on the fraction of viable vs. injured/dead cells and cell numbers. The control sample contained 4 × 10^5^ cells/mL of *Microcystis* and *Dolichospermum* in Lake Champlain water. Viable vs. injured/dead cell concentrations were determined with flow cytometry.

Cultured cells (*Microcystis* and *Dolichospermum*) were spiked into Lake Champlain surface water prior to their exposure to chlorine, ozone, potassium permanganate, and hydrogen peroxide. Oxidant exposures (CTs) were calculated via decay rates according to Equation (1) with the rates and CTs shown in Table S1 of the Supplementary Materials. The CTs for the five chlorine doses ranged from 6.9–36.7 mg-min/L. The two highest ozone exposures were 2.15–2.94 mg-min/L, but given the immediate decay of ozone, doses of 0.5 and 1 mg/L (at 5 min) had nominal CTs of 0 mg-min/L. Potassium permanganate was dosed at 2 and 5 mg/L for 120 min or CTs of 172 and 456 mg-min/L and hydrogen peroxide was dosed at 5 and 10 mg/L for 6 h or CTs of 837 and 2168 mg-min/L. The impact of this oxidation on the fraction of viable vs. injured/dead cells (as determined with flow cytometry) as compared to the cell numbers is shown in Figure 1. Across all oxidants, the control sample initially contained 4 × 10^5^ cells/mL split equally between the *Microcystis* and *Dolichospermum*. 

For chlorine, 24% of the cells remained viable at a CT of 6.9 mg-min/L, whereas the cell count had only dropped to 98%. CTs of 11.7–36.7 mg-min/L all had less than 1% viable cells, but the cell numbers continued to decrease until they reached 26%, at which point 2 × 10^4^ cells/mL of *Dolichospermum* and 7 × 10^4^ cells/mL of *Microcysis* remained (Figure 1). In order to compare results with previous work, a rate of cell lysis was calculated from the cell counting results according to pseudo first-order reaction kinetics (Equation (2)) and found to be 53 M^−1^s^−1^ (R^2^ = 0.91) and 24 M^−1^s^−1^ (R^2^ = 0.90) for *Dolichospermum* and *Microcystis*, respectively. The efficacy of chlorine here was similar to previous work in the both CT required (7–29 mg-min/L) and the lysis rates (30–170 M^−1^s^−1^; pH 7–8.6 with 5–20 × 10^4^ cells/mL in surface waters) with differences attributed to the pH, specific background organic matter, and the presence of mixed species [20,24,29]. 

With ozone doses of 0.5 and 1 mg/L, cell viability was reduced by 10 and 20% and cell numbers decreased to 3.4 and 2.4 × 10^5^ cells/mL, respectively (Figure 1). At 2 mg/L for both 5 and 10 min, the cell numbers decreased to 1 × 10^3^ cells/mL for *Dolichospermum* and 5 × 10^3^ cells/mL for *Microcystis* with the total viable cells <1%. This is in-line with previous work in the absence of background organic matter in which a rapid reduction in cell viability was observed with ozone [17,19,20,21]. Even with potential scavenging from background organic matter, Zamyadi et al. [26] observed that 90% of cells were no longer viable following pre-ozonation (1.2 mg/L, with 20 min of contact time) of a cyanobacterial bloom in a full-scale water treatment plant. 

The two doses of potassium permanganate and hydrogen peroxide saw very limited changes in cell numbers with less than 5% reduction in *Dolichospermum* and *Microcystis*. For potassium permanganate, the flow cytometry results showed that only 67 and 15% of total cells were viable at 172 and 456 mg-min/L, respectively. In previous work, without background organic matter, lower CTs were required to achieve 90% loss of cell viability [18,20]. After dosing with hydrogen peroxide, viable cells were reduced to 35% with 5 mg/L and 27% with 10 mg/L, but the cell counts remained at >90% the original levels. To achieve significant reduction in cell counts, extended contact times and high doses, e.g., > 10 mg/L and 2 days, were required to produce 80% lysis of *Microcystis* [18,37]. 

The discrepancy between the reduction in cell numbers (generated from cell counting) vs. cell-viability (determined via flow-cytometry) was significantly smaller after ozone relative to chlorine, potassium permanganate and hydrogen peroxide. This is reflective of the type of damage imparted by the oxidant as well as exposure dose. SEM was used to generate images of cells before and after oxidation with ozone, chlorine, and hydrogen peroxide (Figures S1 and S2). Though a very limited data set, these images provide insight into the impact of chlorine, ozone, and hydrogen peroxide on the morphology. Under chlorine (37 mg-min/L) and ozone (0.5 mg/L, 5 min), the cell links between *Dolichospermum* cells were the first part of the cell damaged whereas hydrogen peroxide (837 mg-min/L) shrank and deformed the cells, but fragmentation of the filaments did not occur. *Microcystis* cells appeared deformed under ozone and chlorine, but not fragmented. These results are similar to those observed in past studies with SEM where ozone and chlorine produced a similar breakup of filaments and deformation of cells [19,38]. 

As shown with flow cytometry, cell counting and SEM images, ozone produced fragmentation and the release of intracellular material, but potassium chlorine, permanganate and hydrogen peroxide damaged cell walls and viability. This result was further supported by the negligible (<0.1 mg/L) releases of dissolved organic carbon (DOC) after oxidation with potassium permanganate and hydrogen peroxide relative to the 0.55 mg/L released after chlorination (max across all CTs) and 1.26 mg/L after ozonation (2 mg/L with 5 or 10 min contact times). 

### 2.2. LC-OCD-OND

**Figure 2:**
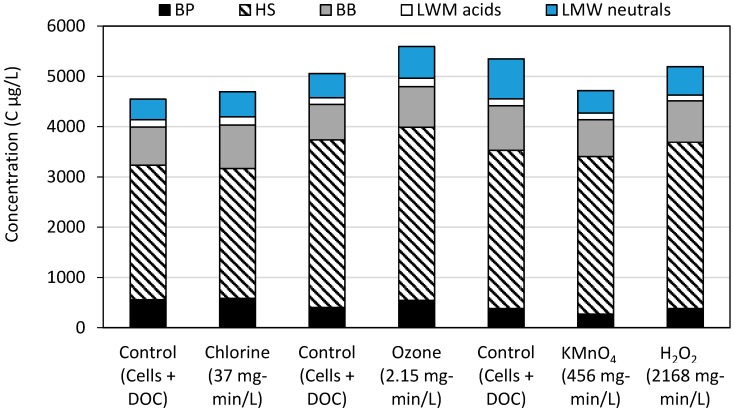
Organic matter fractions before (control) and after the oxidation of *Microcystis* and *Dolichospermum* in Lake Champlain water. The three different control samples are the same matrix, conducted as separate experiments. Components were identified via LC-OCD-OND based on Huber et al. [41]: BB = building blocks; HS = humic substances; BP = Biopolymer; LMW = low molecular weight.

To evaluate the changes in DOC induced by oxidation, the organic matter fractions were determined with LC-OCD-OND. Although cell viability was monitored at multiple CTs, the max CT for each oxidant was used for LC-OCD-OND, i.e., chlorine at 37 mg-min/L, ozone at 2.15 mg-min/L, potassium permanganate at 456 mg-min/L, and hydrogen peroxide at 2168 mg-min/L. From the OCD peaks, the concentration of biopolymers, humic substances, building blocks, low molecular weight (LMW) acids, and LMW neutrals were determined (Figure 2 and Figure S4). Biopolymers include high molecular weight (>10 kDa) polysaccharides, proteins, amino acids, and extracellular polymeric substances. OND provided information regarding the nitrogen content of a subset of the DOC including biopolymer DON, biopolymer N/C ratio, humic substances DON and humic substances N/C ratio. Based on the UVD and the OCD signal, the specific UV absorption (SUVA) at 254 nm of humic substances was also determined (Table S2). Algal organic matter is generally divided into intracellular organic matter (IOM) and extracellular organic matter (EOM). IOM is primarily composed of proteins, polysaccharides, lipids and humic-like substances [16,39] and Henderson et al. [40] showed that the EOM of *Microcystis* contains primarily hydrophilic polysaccharides and proteins. 

The control sample contained both the background organic matter from Lake Champlain and any cell fragments able to pass through the 0.45 μm filter (Figure 2). Across all oxidants, humic substances SUVA_254_ values were lower after exposure due to oxidation reactions with the aromatic sites [42] (Table S2). Decreases of the number-averaged molecular weight of humic substances also occurred across all waters from the breakdown of organic matter present and in the case of ozone, the integration of oxygen resulting in more soluble components [43] (Table S2). The humic substances fraction generally saw minimal change (<5%) after oxidation. This was likely due to the background organic matter, which makes up the majority of this fraction and would have been oxidized, but not mineralized at these doses. 

Ozonation increased the fraction of humic substances by 3%, biopolymers by 36%, building blocks by 15%, LMW acids by 28% and LMW neutrals by 31% (Figure 2). Chlorination also increased these fractions, though to a lower extent at 4.8% for biopolymers, 14% for building blocks, 13% for LMW acids, and 20% for LMW neutrals. Biopolymers likely increased due to the release of IOM, but they may also have come from fragments of the cell membrane or extracellular material (e.g., amino acids, polysaccharides and proteins) [39]. The DON results supported this conclusion as ozonation also increased the concentration of DON biopolymers from 56 to 216 ppb-N and the ratio of N/C from 0.14 to 0.40. An important caveat to these results is that the formation of building blocks and LMW acids/neutrals could also have come from the degradation of humic substances. 

Potassium permanganate and hydrogen peroxide produced the degradation of all fractions (biopolymer, building blocks, LMW acids/neutrals) and a minor loss of the total carbon, which was attributed to biological consumption during the reaction period (Figure 2). However, DON biopolymers increased in concentration from 10 to 54 ppb-N (after H_2_O_2_) and 26 ppb-N (after KMnO_4_) indicative of select IOM release not detected in the bulk DOC. These releases coincided with a change in the fraction of N/C from 0.03 in the control to 0.09 after potassium permanganate and to 0.14 after hydrogen peroxide indicative of the presence of added material from biomass. 

The LC-OCD-OND results helped to identify the fractions found in EOM and IOM, which are potential components that could scavenge oxidants and hinder the effective modeling of the oxidation of cyanotoxin and T&O compounds. Although these fractions have been analyzed as a potential source of disinfection byproducts and interference to coagulants, they have only been included in a limited set of competition kinetic studies wherein potassium permanganate was scavenged by DOC during the oxidation of cyanotoxins [44]. 

### 2.3. EDM/HSI

**Figure 3:**
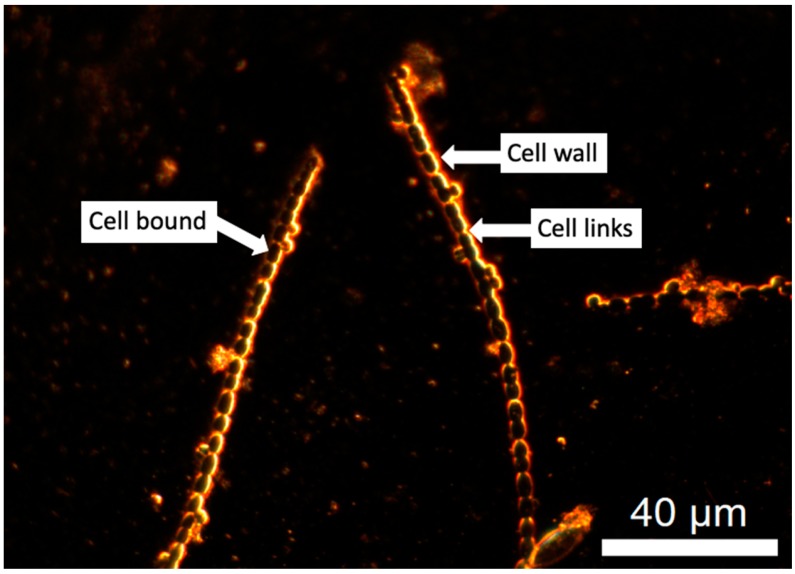
EDM image of *Dolichospermum* before oxidation, illustrating different pixels targeted for spectral analysis: intracellular or cell-bound material, cell links, and cell wall. The EDM image for *Microcystis* with labels for intracellular or cell-bound material and cell wall is shown in Figure S3.

The flow cytometry, SEM, and LC-OCD-OND results were compared against the spectra generated with EDM/HSI. EDM/HSI allows for spectra (400–1000 nm) to be generated from a specific pixel (containing a cell component of interest). In this case, the components of interest for oxidation were the cell wall and cell-bound material for *Microcystis* and cell wall, cell-bound material, and the links between cells for *Dolichospermum*. Figure 3 (*Dolichospermum*) and Figure S3 (*Microcystis*) are labeled EDM images that show examples of where the cell component spectra were collected. During the experiments, three pixels were selected from each cell component to minimize cell specific variability. The collected spectra were smoothed using a moving average to minimize the noise within the spectra and signal responses were normalized to the maximum value within each spectrum for comparison. EDM/HSI was only conducted for samples oxidized with chlorine (CT of 37 mg-min/L) and ozone (0.5 mg/L for 5 min). After this chlorine exposure (37 mg-min/L), >99% of cells were not viable and only 26% of total cells remained from cell counting. The 0.5 mg/L and 5 min ozone exposure resulted in a 20 and 40% reduction in the viable cells and cell numbers, respectively.

**Figure 4:**
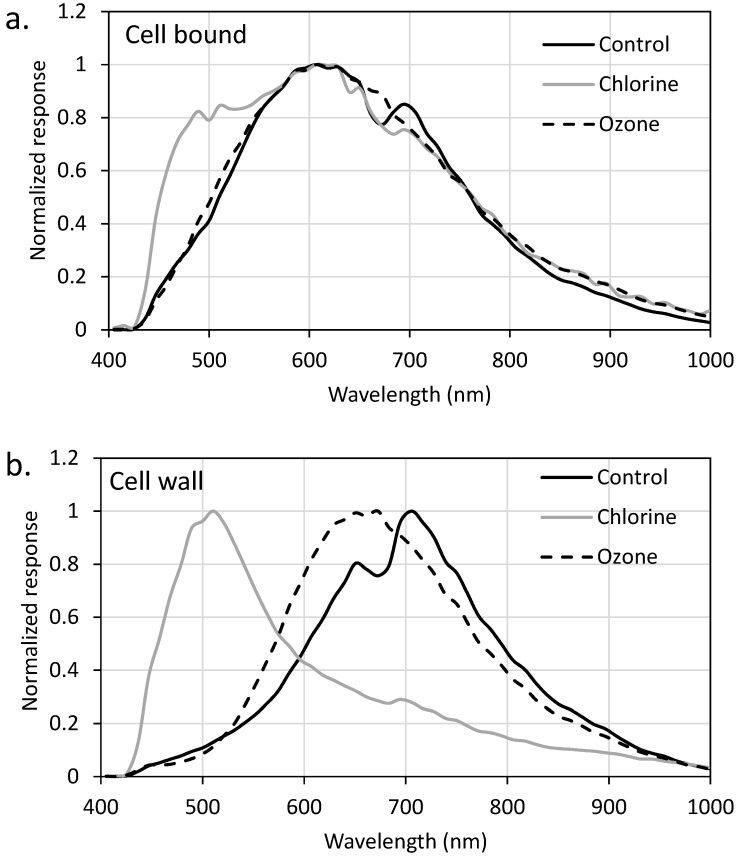
Impact of chlorine (CT = 37.5 mg-min/L) and ozone (0.5 mg/L, 5 min) on *Microcystis* (**a**) cell-bound material and (**b**) cell wall. EDM was used to find a pixel containing only the cell-bound material or cell wall where HSI spectra were collected. Instrument responses were normalized to the maximum value of each spectra for comparison.

The two species had uniquely shaped spectra regardless of cell component (Figures S5 and S6). However, the specific cell components shared common peaks/shoulders for both species, even if the peaks were slightly higher or lower in their maximum wavelength. For *Microcystis* cells, the spectra had two peaks at approximately 600–650 and 705–715 nm. *Dolichospermum* had three main features with two peaks at 650–665 and 695–705 nm and a shoulder at 530–550 nm. For *Microcystis*, the cell-bound material did not show a dramatic change following chlorination and ozonation (Figure 4a). The ozonated cell-bound material was still dominated by one peak at ~620 nm and after chlorination, the two cell-bound peaks remained, but a shoulder emerged centered around ~500 nm. In contrast to the cell-bound material, the cell wall spectra were significantly impacted by the two oxidants, showing that both oxidants impact the cell membrane before other components of the cell (Figure 4b). The ozonated cell wall retained a single peak at 660 nm, though significantly broader than the control. The chlorinated cell wall produced a new peak at ~500 nm and the removal of the two peaks originally exhibited by the control sample at ~660 and ~715 nm. 

**Figure 5:**
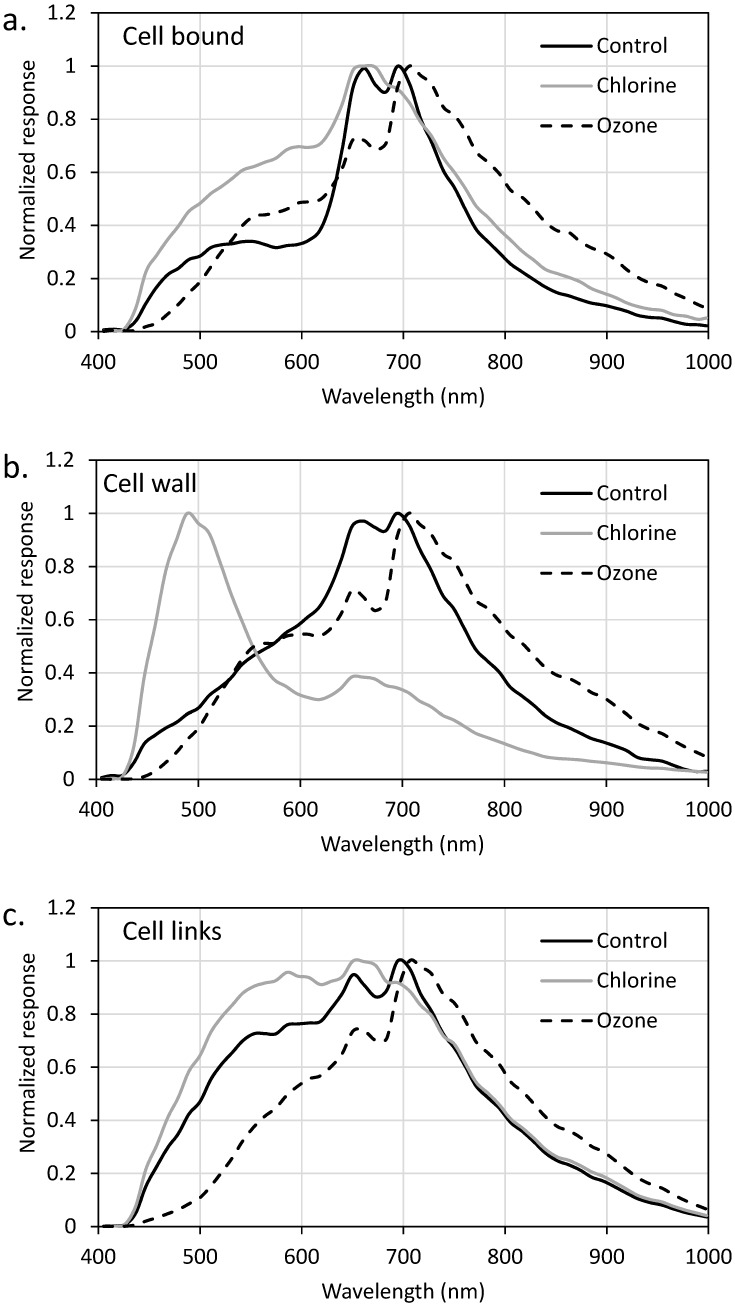
Impact of chlorine (CT = 37.5 mg-min/L) and ozone (0.5 mg/L, 5 min) on *Dolichospermum* (**a**) cell-bound material, (**b**) cell wall, and (**c**) cell links. EDM was used to find a pixel containing only the cell-bound material, cell wall, or links between cells where HSI spectra were collected. Instrument responses were normalized to the maximum value of each spectra for comparison.

SEM images of *Dolichospermum* cells showed that the cell links were targeted first by ozone and chlorine, but the EDM/HSI spectra did not show this result. When the cell links were oxidized (Figure 5c), the ozonated spectra were very similar to the control sample with the same peaks observed. The chlorination of the cell links produced a new shoulder with a max peak around 595 nm, but it smoothed the two other peaks observed in the control. The ozonated cell-bound material retained the two peaks of the control, though the ~660 nm peak was lower than the ~700 nm peak; the control shoulder remained, though shifted to lower energy wavelengths (~550nm at max). Chlorination had peaks at ~660 and ~700 nm, and the shoulder remained intact with a max peak at ~590 nm. For the cell wall, the ozonated water response mirrored those observed in the control at ~520 nm (shoulder), ~660 nm (peak), and ~700 nm (peak). As was observed with *Microcystis*, chlorine resulted in the formation of a peak centered at ~500 nm, a peak at ~660 nm and the removal of the peak at ~700 nm. 

The presence of all the peaks identified within these culture samples were likely due to the overlapping absorption of pigments within the cells (e.g., phycocyanin, chlorophyll). The shifts in their absorption to a single peak would appear to indicate a change in the pigments present within the cell wall, cell links or cell-bound material as a result of oxidation. In remote sensing applications, HSI peaks of 620 and 665 nm were found to corelate with on-the ground levels of phycocyanin and chlorophyll-*a*, respectively [34,35]. The ~700 nm peak is not used in remote sensing applications because of the interference from water; this interference comes from the application of HSI in reflectance mode as opposed to transmittance mode, which is typically applied at the bench-scale [35]. The differences in spectra shape have been taken advantage of to differentiate between *Aphanizomenon flos-aqua* and *Microcystis* at the bench-scale [33], but this was accomplished with a spectral shape algorithm applied to the derivative of the spectrum to obtain meaningful results, which is beyond the scope of this work.

## 3. Conclusions

Flow cytometry and cell counting after oxidation with ozone, chlorine, potassium permanganate and hydrogen peroxide highlighted the discrepancy between cell viability vs. cell lysis and fragmentation. These differences were also observed visually via SEM, which showed the shrinking/deformation of *Microcystis* and *Dolichospermum*, but not complete lysis. SEM results also showed the fragmentation of *Dolichospermum* cell links prior to cell lysis, which has been observed in previous work [19,30,38]. 

LC-OCD-OND was used to identify the fractions of IOM and EOM released after the oxidation of *Microcystis* and *Dolichospermum* in addition to the changes in bulk organic matter from Lake Champlain. Following ozonation and chlorination, increases in the biopolymer fraction were attributed to the release of IOM and potentially fragments of the cell membrane (e.g., amino acids, polysaccharides, and proteins). Future work should aim to better understand how this fraction of released IOM and oxidized EOM impacts the degradation of compounds of interest, e.g., cyanotoxins and T&O compounds. 

EDM/HSI revealed the unique spectra of both the *Dolichospermum* and *Microcystis*. Both cultures had spectra with peaks at ~660 and ~700 nm, but *Dolichospermum* had a shoulder with a peak at ~550 nm. The oxidation of these cells shifted the responses, which was attributed to the decrease in concentration of pigments present. Both cell wall spectra revealed the formation of a peak after chlorination at ~500 nm, but limited changes were observed following ozonation. 

## 4. Materials and Methods 

### 4.1. Cyanobacteria Culture Sample Preparation

*Microcystis aeruginosa* strain CPCC 300 (referred to as *Microcystis* in the manuscript) and *Anabaena sp.* strain CPCC 544 (referred to as *Dolichospermum* in the manuscript due to recent nomenclature changes) were cultured in BG-11 medium. Cultures were incubated at 21 °C under a 12-h rotating light–dark cycle at an intensity of 70 µmol S^−1^ m^−2^. Cultures were harvested at stationary phase and spiked into water from Lake Champlain to reach a concentration of 4 × 10^5^ cells/mL. The Lake Champlain sample was collected from a water treatment plant intake in southern Quebec, Canada in late October and early November of 2018. During this time, the utility measured its intake as having 5–6 mg/L DOC and a pH of 6.5–7.

### 4.2. Preparation of Oxidants and Calculation of Exposure 

A 2000-mg/L free chlorine stock solution was prepared from sodium hypochlorite (5.25%) on the day of the experiment. The N,N-diethyl-p-phenylenediamine (DPD) colorimetric method was used to measure free chlorine concentration according to Standard Methods (SM) 4500-Cl G [45]. Chlorine doses of 1, 2, and 3 mg/L were added to the two cultures and exposed for 10, 20 and 30 min of contact time. Samples were exposed at room temperature (22 °C) and chlorine residual measurements were taken to estimate oxidant exposure. Samples were quenched sodium thiosulfate at a dose of 1.1 mg/L per 1 mg/L chlorine from a stock solution of 3000 mg/L. 

An ozone stock solution (50–60 mg/L) was prepared with gaseous ozone using a bench-scale ozone generator (additional details in Zamyadi et al. [21]). Ozone stock concentration and residual ozone in water samples were measured using SM 4500-O_3_ [45]. Ozonation experiments were conducted with doses of 0.5, 1, and 2 mg/L with 5 and 10 min contact time. Again, samples were quenched with 1.6 mg/L sodium thiosulfate per 1 mg/L ozone. Due to the rapid decay of ozone, residuals were below detection for the 0.5 and 1 mg/L doses. 

Potassium permanganate (KMnO_4_) crystals were dissolved in ultrapure water to prepare a stock solution of 5000 mg/L. Experiments were conducted with 2 and 5 mg/L with 120 min contact time. Samples were taken at specific times to measure potassium permanganate residual and measured according to the DPD colorimetric method ratio of 0.891 KMnO_4_/Cl_2_ SM 4500-Cl G [45]. After 120 min of contact time, 1.2 mg/L sodium thiosulfate per 1 mg/L KMnO_4_ was used to quench further oxidation.

Hydrogen peroxide (H_2_O_2_) stock (10 g/L) was prepared from a purchased solution of stabilized hydrogen peroxide (30%). Cyanobacteria suspensions were dosed with 5 and 10 mg/L of hydrogen peroxide and exposed for 6 h of contact time. The hydrogen peroxide residual was measured using a colorimetric test kit (Chemetrics K-5510, Midlands, VA, USA). After the contact time, the residual was quenched with 1.2 mg/L of sodium thiosulfate per 1 mg/L H_2_O_2_.

Oxidant exposures (CT) were calculated using Equation (1):

(1) $$CT=\int_{0}^{t}\left[Oxidant\right]dt=\frac{C_{0}}{k_{decay}}(e^{k_{decay}t}-1)$$

where *k_decay_* (min^−1^) is the first-order decay rate, *t* (min) is the exposure time, and *C*_0_ (mg/L) is the initial concentration of oxidant at time zero. CTs for chlorine, ozone, potassium permanganate, and hydrogen peroxide are shown in the Table S1. 

CTs were subsequently used to calculate the cell lysis rate:

(2) $$C=C_{0}\times e^{-kR}$$

where *C* was concentration [total cell number (cells/mL)] at a given time, *C*_0_ was the starting concentration [cell number (cells/mL) prior to oxidant exposure], *k* was the rate [M^−1^s^−1^], and *R* was the oxidant exposure [CT (mg-min/L)]. 

### 4.3. DOC and LC-OCD-OND

DOC and LC-OCD-OND samples were filtered via a pre-rinsed 0.45-µm membrane (Supor 45 µm, 47 m, PES PALL, Port Washington, NY, USA) and stored in carbon-free glass vials. A 5310 total organic carbon analyzer (Sievers Analytical Instruments, Boulder, CO, USA) was used to measure DOC. LC was coupled with OCD, OND, and UVD (254 nm) to track the DOC, DON, and UV responses over chromatographic retention times with various peaks identified and integrated according to Huber et al. [41]. From the LC-OCD, the concentrations of biopolymers (e.g., high molecular weight (>10 kDa) polysaccharides, proteins, amino acids, and other components in extracellular polymeric substance), humic substances, building blocks (e.g., low molecular weight humic substances), low molecular weight (LMW) acids, and LMW neutrals were determined. An example chromatogram with labeled peaks can be found in Figure S4. The added data from LC-OND and UVD provided the concentration of biopolymer DON and N/C ratio as well as humic substances DON, N/C ratio, and specific UV absorption (SUVA) at 254 nm. 

### 4.4. Cell Counts, Morphology and Integrity

Cell counts were determined using an inverted microscope in a Sedgwick–Rafter chamber with 20× magnification on cells preserved with Lugol’s Iodine [46,47]. Images of cyanobacteria during oxidation were captured using a SEM (JSM-7600 TFE, JEOL, Akishima, Japan) according to the methods applied in [19]. Cell integrity was determined using flow cytometry (BD Accuri C6 Flow Cytometer, San Jose, CA, USA) and samples were stained with SYBR Green I (SG) and SG propidium iodide to determine total and compromised/dead cells [30,48]. A pre-test with pure cultures (*Microcystis* and *Dolichospermum*) was performed to define a suitable gating to differentiate cyanobacteria cells from other bacteria. This modified gating was used in all subsequent flow cytometry analysis. 

Sample morphology was analyzed under optical microscope equipped with darkfield illumination and hyperspectral analysis or EDM/HSI (CytoViva, Auburn, AL, USA) [49]. HSI was equipped to monitor wavelengths from 400–1000 nm for every pixel of the image, which was captured at 40× magnification. Pixel sizes were estimated to be 265 × 265 nm. Quenched samples were put on a microscope slide (500 µL) and then dried under the fume hood to avoid the movement of liquid during EDM/HSI. A spot on the slide containing both cyanobacteria species (*Microcystis* and *Dolichospermum*) was selected to capture the image and acquire spectral data.

## Supplementary Materials

The following are available online at https://www.mdpi.com/2072-6651/11/5/278/s1, Figure S1: SEM images of the cyanobacteria morphology both before and after chlorination (CT of 37.5 mg-min/L): (**a**) *Microcystis* in control (3000×) (**b**) chlorinated *Microcystis* cells (8500×), (**c**) *Dolichospermum* cells in control (1400×), and (**d**) chlorinated *Dolichospermum* cells (1600×). Figure S2: SEM image of cyanobacteria after (**a**) ozonation of both *Microcystis* and *Dolichospermum* (0.5 mg/L, 5 min exposure at 4000×) and (**b**) hydrogen peroxide application on *Dolichospermum* (837 mg-min/L at 2200×). Figure S3: EDM image of un-oxidized *Microcystis* with cell wall and intracellular material identified. Figure S4: LC-OCD chromatogram of the un-oxidized control cyanobacteria sample. *Microcystis* and *Dolichospermum* were spiked into Lake Champlain water and filtered (0.45 μm). Figure S5: HSI responses of cell-bound material, cell wall, and cell links for *Dolichospermum*. Instrument responses were normalized to the maximum value of each spectra for comparison. Figure S6: HSI responses of cell-bound material and cell wall for *Microcystis*. Instrument responses were normalized to the maximum value of each spectra for comparison. Table S1: First-order decay rates for chlorination and resulting CT. Table S2: Impact of oxidation on organic carbon fractions with LC-OCD-OND-UVD.
