# Supplementary material for: Using Advanced Spectroscopy and Organic Matter Characterization to Evaluate the Impact of Oxidation on Cyanobacteria
Source: Toxins (Basel). 2019 May 17;11(5):278. doi: 10.3390/toxins11050278 (PMC6563301; doi:10.3390/toxins11050278)
Supplement: Supplementary file 1 [file toxins-11-00278-s001.pdf]

Supplementary Materials: Using Advanced Spectroscopy and Organic Matter Characterization to Evaluate the Impact of Oxidation on Cyanobacteria

Saber Moradinejad, Caitlin M. Glover, Jacinthe Mailly, Tahere Zadfathollah Seighalani, Sigrid Peldszus, Benoit Barbeau, Sarah Dorner, Michèle Prévost and Arash Zamyadi

Table S1. First-order decay rates for chlorination and resulting CT.

Oxidant Dose and Contact Time		k (min^{-1})	R^2	CT (mg-min/L)
**Chlorine**	1 mg/L, 10 min	1.43×10^{-5}	0.98	5.2
	1 mg/L, 20 min	1.36×10^{-5}	0.99	6.9
	2 mg/L, 10 min	1.18×10^{-5}	0.95	11.7
	2 mg/L, 20 min	7.1×10^{-3}	0.98	21.4
	2 mg/L, 30 min	6.7×10^{-3}	0.99	21.7
	3 mg/L, 10 min	6.8×10^{-3}	0.92	25.9
	3 mg/L, 20 min	6.3×10^{-3}	0.98	35.9
	3 mg/L, 30 min	7.0×10^{-3}	0.94	37.5
**Ozone**	2 mg/L, 5 min	8.64×10^{-3}	0.84	2.94
	2 mg/L, 10 min	1.70×10^{-3}	0.94	2.15
**$KMnO_4$**	2 mg/L, 120 min	9.89×10^{-5}	0.98	172
	5 mg/L, 120 min	7.99×10^{-5}	0.98	456
**H_2O_2**	5 mg/L, 6 hr	8.29×10^{-5}	0.95	837
	10 mg/L, 6 hr	5.17×10^{-5}	0.99	2168

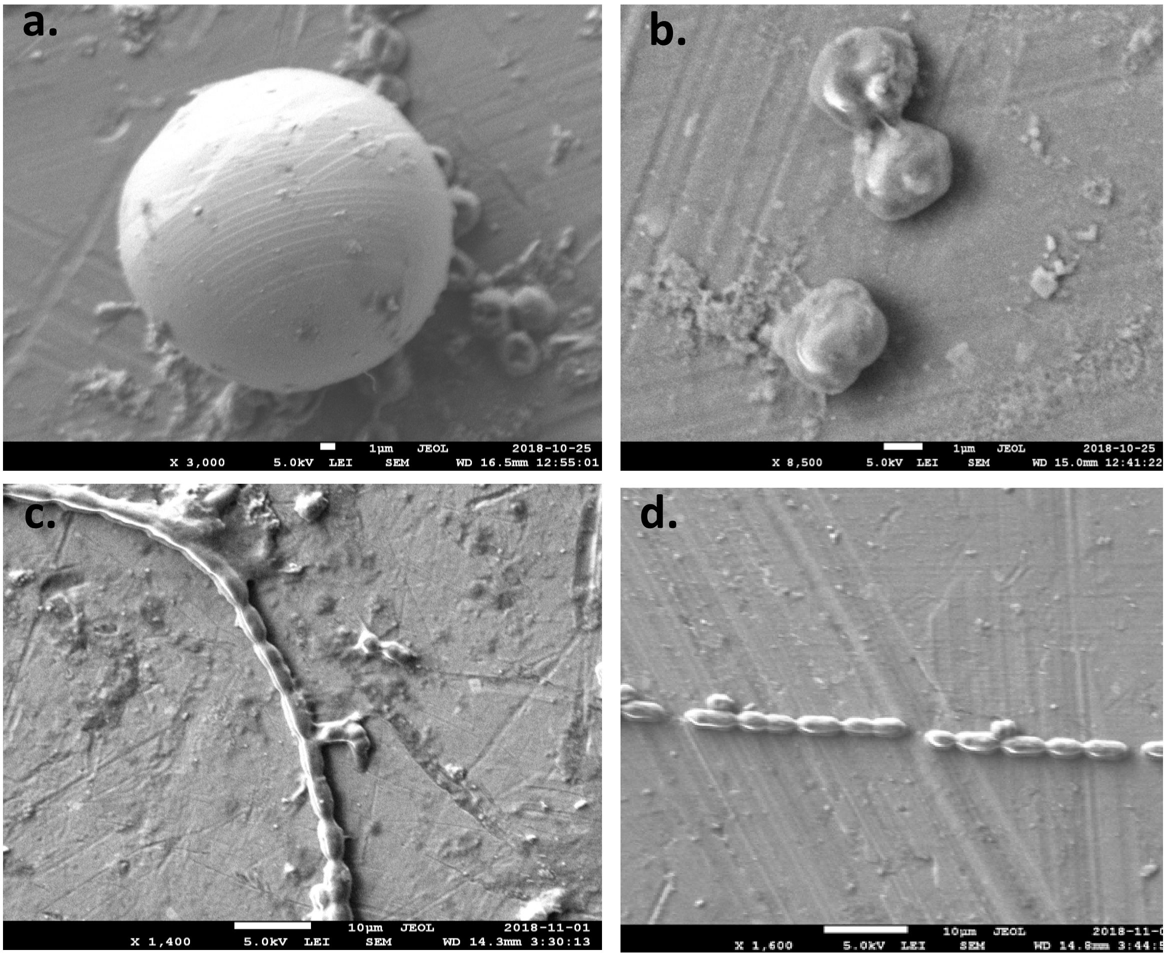

Figure S1. SEM images of the cyanobacteria morphology both before and after chlorination (CT of 37.5 mg-min/L): **a**) *Microcystis* in control (3000×) **b**) chlorinated *Microcystis* cells (8500×), **c**) *Dolichospermum* cells in control (1400×), and **d**) chlorinated *Dolichospermum* cell (1600×).

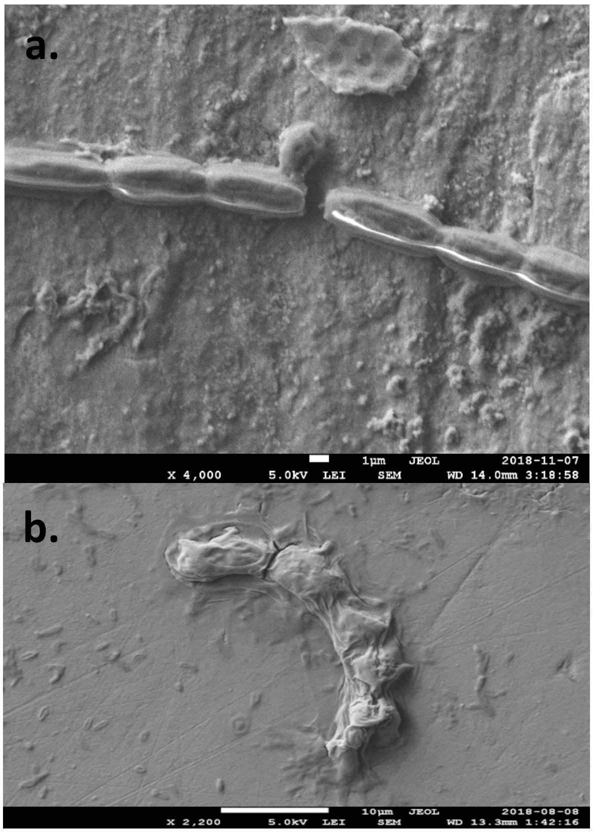

Figure S2. SEM image of cyanobacteria after **a**) ozonation of both *Microcystis* and *Dolichospermum* (0.5 mg/L, 5 min exposure at 4000×) and **b**) hydrogen peroxide application on *Dolichospermum* (837 mg-min/L at 2200×).

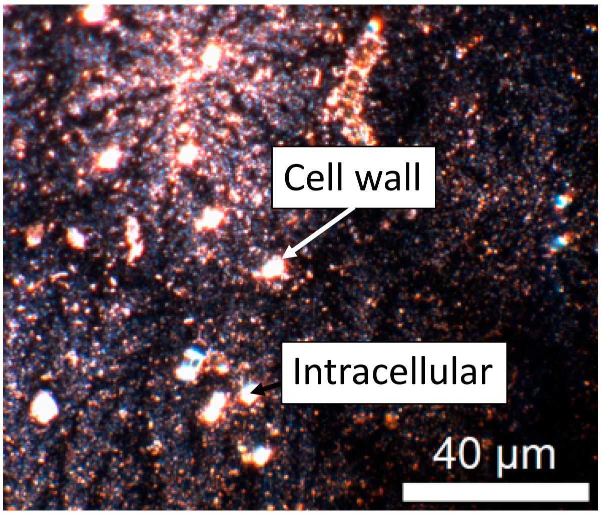

Figure S3. EDM image of un-oxidized *Microcystis* with cell wall and intracellular material identified.

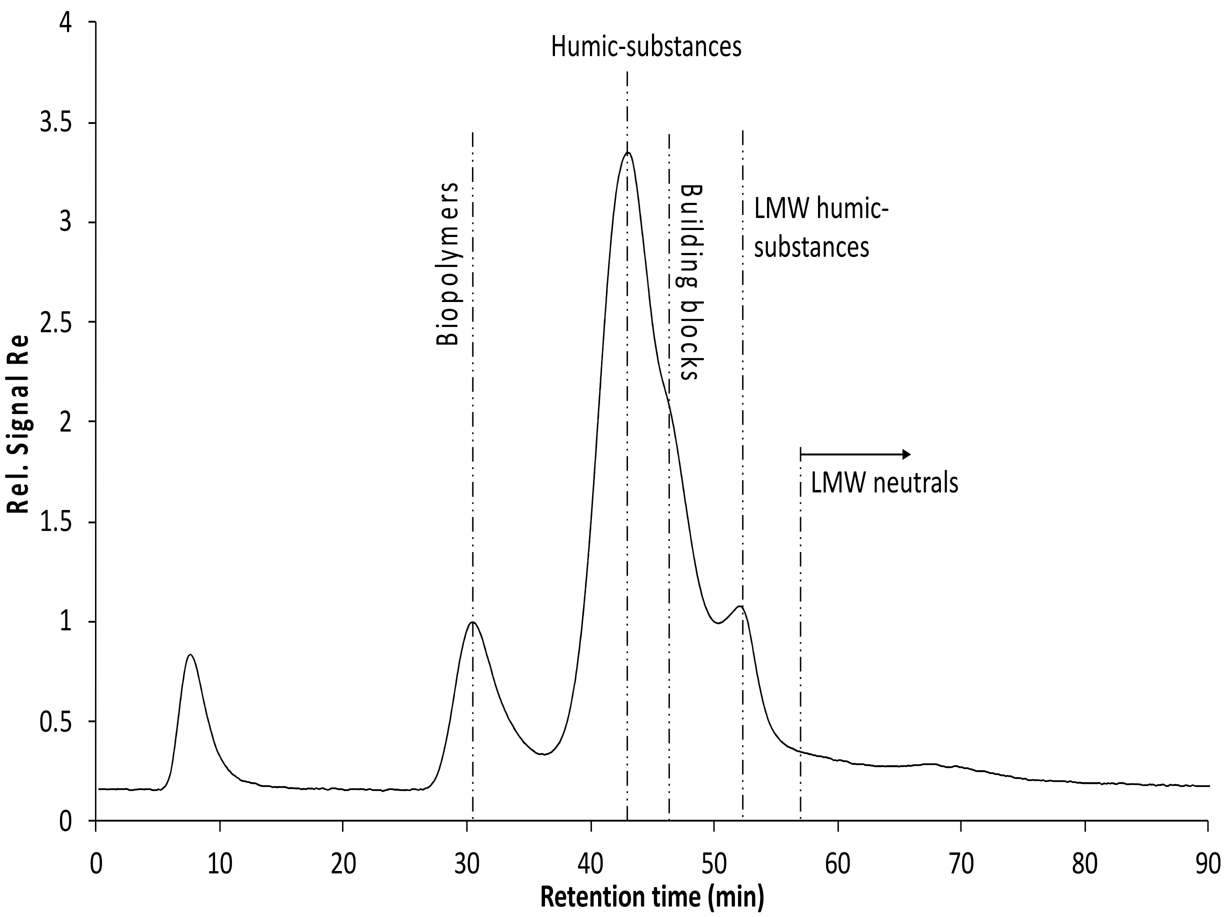

Figure S4. LC-OCD chromatogram of the un-oxidized control cyanobacteria sample. *Microcystis* and *Dolichospermum* were spiked into Lake Champlain water and filtered (0.45 µm).

Table S2. Impact of oxidation on organic carbon fractions with LC-OCD-OND-UVD.

Sample	Concentration (µg C/L)					
	**Total DOC**	**Bio-Polymer**	**Humic-Substances**	**Building Blocks**	**LMW Acids**	**LMW Neutrals**
Chlorine control	5184	556	2677	757	145	411
Chlorine (3 mg/L, 30 min)	5216	583	2583	866	145	496
Ozone control	5632	402	3335	706	131	481
Ozone (2 mg/L, 10 min)	6382	547	3440	810	167	630
$KMnO_4$ and H_2O_2 control	6134	383	3146	884	140	796
KMnO4 (5 mg/L, 120 min)	5533	271	3131	735	131	448
H_2O_2 (5 mg/L, 6 hr)	5913	380	3308	825	114	565

Sample	Bio-Polymer DON (μg N/L)	Bio-Polymer N/C (μg/μg)	Humic-Substances DON (μg N/L)	Humic-Substance N/C (μg/μg)	Humic-Substances SUVA (L mg^{-1}m^{-1})	Humic-Substances Molecular Weight (M_n-g/mol)
Chlorine control	61	0.11	147	0.05	3.93	624
Chlorine (3 mg/L, 30 min)	47	0.08	133	0.05	3.25	626
Ozone control	56	0.14	136	0.04	3.99	626
Ozone (2 mg/L, 10 min)	216	0.40	140	0.04	2.84	583
$KMnO_4$ and H_2O_2 control	13	0.03	108	0.03	4.45	701
KMnO4 (5 mg/L, 120 min)	21	0.09	101	0.03	3.93	638
H_2O_2 (5 mg/L, 6 hr)	54	0.14	121	0.04	4.34	570

LMW = low molecular weight. Building blocks = low molecular weight humic-like substances. Biopolymer = high molecular weight (>10 kDa) polysaccharides, proteins, amino-acids, and other components in extracellular polymeric substances.

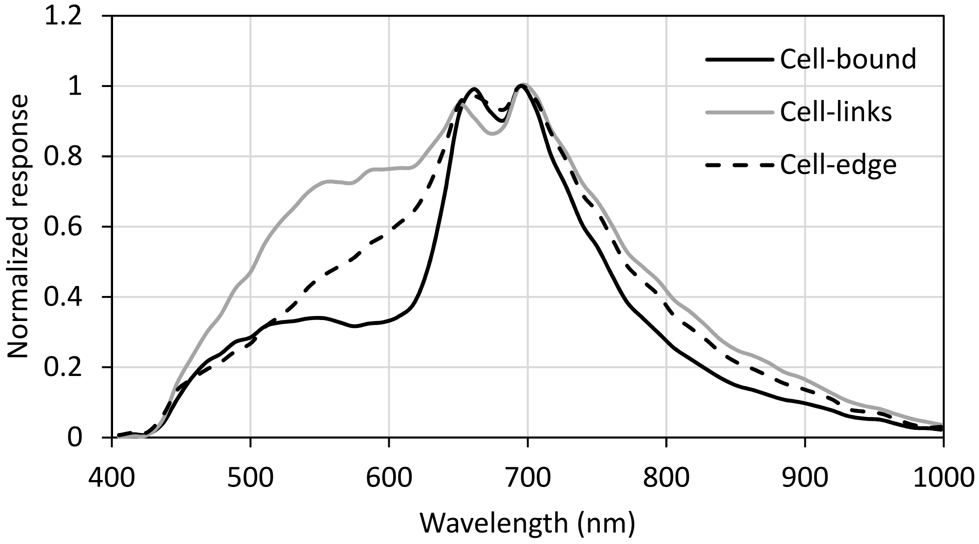

Figure S5. HSI responses of cell-bound, cell-wall, and cell-links for *Dolichospermum*. Instrument responses were normalized to the maximum value of each spectra for comparison.

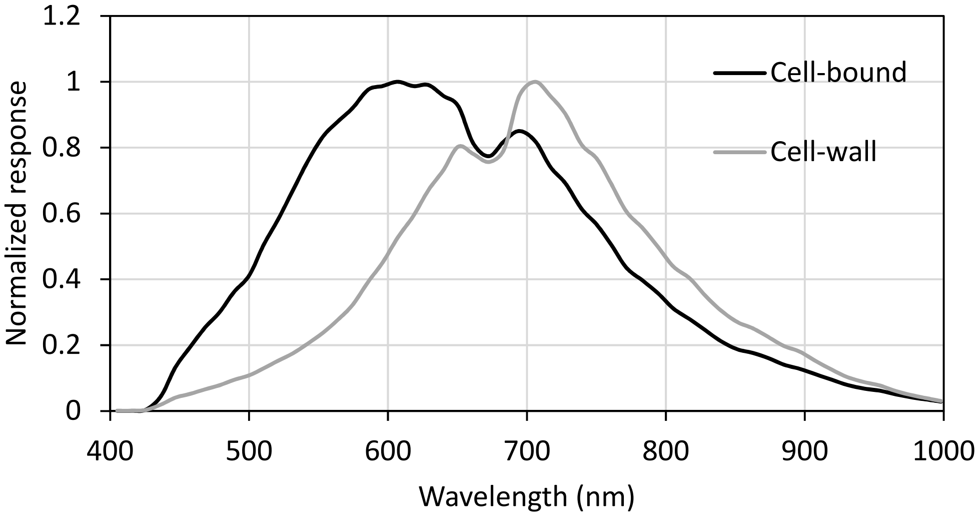

Figure S6. HSI responses of cell-bound and cell-wall for *Microcystis*. Instrument responses were normalized to the maximum value of each spectra for comparison
